# (*E*)-4-Bromo-*N*′-(2-nitro­benzyl­idene)benzohydrazide

**DOI:** 10.1107/S1600536809002165

**Published:** 2009-02-11

**Authors:** Ming-Jun Zhang, Li-Zi Yin, Da-Cheng Wang, Xu-Ming Deng, Jing-Bo Liu

**Affiliations:** a College of Animal Science and Veterinary Medicine, Jilin University, Changchun 130062, People’s Republic of China; b Laboratory of Nutrition and Functional Foods, Jilin University, Changchun 130062, People’s Republic of China

## Abstract

The title compound, C_14_H_10_BrN_3_O_3_, was obtained by a condensation reaction between 2-nitro­benzaldehyde and 4-bromo­benzohydrazide. The dihedral angle between the two benzene rings is 4.1 (2)°. The mol­ecule displays an *E* configuration about the C=N bond. In the crystal, mol­ecules are linked into a chain along [100] by inter­molecular N—H⋯O hydrogen bonds.

## Related literature

For the biological properties of Schiff base and hydrazone compounds, see: Kucukguzel *et al.* (2006 ▶); Khattab *et al.* (2005 ▶); Karthikeyan *et al.* (2006 ▶); Okabe *et al.* (1993 ▶). For bond-length data, see: Allen *et al.* (1987 ▶). For related structures, see: Shan *et al.* (2008 ▶); Fun *et al.* (2008 ▶); Ma *et al.* (2008 ▶); Diao *et al.* (2008*a* ▶,*b* ▶); Ejsmont *et al.* (2008 ▶).
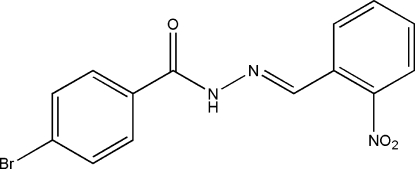


## Experimental

### 

#### Crystal data

C_14_H_10_BrN_3_O_3_
*M* *_r_* = 348.16
Triclinic, 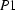

*a* = 4.8718 (17) Å
*b* = 6.842 (2) Å
*c* = 10.709 (4) Å
α = 98.014 (5)°
β = 93.258 (6)°
γ = 97.413 (5)°
*V* = 349.5 (2) Å^3^
*Z* = 1
Mo *K*α radiation
μ = 2.95 mm^−1^
*T* = 298 (2) K
0.20 × 0.18 × 0.17 mm

#### Data collection

Bruker SMART CCD area-detector diffractometer
Absorption correction: multi-scan (*SADABS*; Bruker, 2001 ▶) *T*_min_ = 0.590, *T*_max_ = 0.634
2347 measured reflections
1998 independent reflections
1584 reflections with *I* > 2σ(*I*)
*R*_int_ = 0.013

#### Refinement

*R*[*F* ^2^ > 2σ(*F* ^2^)] = 0.027
*wR*(*F* ^2^) = 0.064
*S* = 0.95
1998 reflections
193 parameters
3 restraints
H atoms treated by a mixture of independent and constrained refinement
Δρ_max_ = 0.22 e Å^−3^
Δρ_min_ = −0.23 e Å^−3^
Absolute structure: Flack (1983 ▶), 235 Friedel pairs
Flack parameter: 0.021 (8)

### 

Data collection: *SMART* (Bruker, 2007 ▶); cell refinement: *SAINT* (Bruker, 2007 ▶); data reduction: *SAINT*; program(s) used to solve structure: *SHELXTL* (Sheldrick, 2008 ▶); program(s) used to refine structure: *SHELXTL*; molecular graphics: *SHELXTL*; software used to prepare material for publication: *SHELXTL*.

## Supplementary Material

Crystal structure: contains datablocks global, I. DOI: 10.1107/S1600536809002165/ci2760sup1.cif

Structure factors: contains datablocks I. DOI: 10.1107/S1600536809002165/ci2760Isup2.hkl

Additional supplementary materials:  crystallographic information; 3D view; checkCIF report

## Figures and Tables

**Table 1:** Hydrogen-bond geometry (Å, °)

*D*—H⋯*A*	*D*—H	H⋯*A*	*D*⋯*A*	*D*—H⋯*A*
N1—H1⋯O1^i^	0.900 (11)	1.909 (19)	2.791 (3)	166 (6)

Symmetry code: (i) 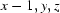.

**Fig. 1.**
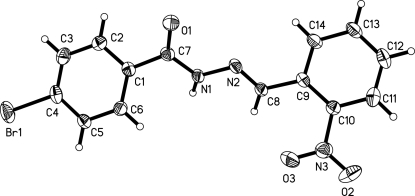
The molecular structure of the title compound with 30% probability displacement ellipsoids for non-H atoms.

## supplementary crystallographic information

### Comment

Hydrazones and Schiff bases have attracted much attention for their excellent biological properties, especially for their potential pharmacological and antitumor properties (Kucukguzel *et al.*, 2006; Khattab *et al.*, 2005; Karthikeyan *et al.*, 2006; Okabe *et al.*, 1993). Recently, a large number of hydrazone derivatives have been prepared and structurally characterized (Shan *et al.*, 2008; Fun *et al.*, 2008; Ma *et al.*, 2008; Diao *et al.*, 2008*a*,b; Ejsmont *et al.*, 2008). As part of the ongoing study, we report herein the crystal structure of the title compound.

The molecular structure of the title compound is shown in Fig. 1. The dihedral angle between the two benzene rings is 4.1 (2)°. The molecule of the compound displays an *E* configuration about the C═N bond. The bond values are typical (Allen *et al.*, 1987). The molecules are linked into a chain along the [100] by intermolecular N—H···O hydrogen bonds.

### Experimental

2-Nitrobenzaldehyde (1.0 mmol, 151.1 mg) was dissolved in methanol (50 ml) and then 4-bromobenzohydrazide (1.0 mmol, 215.0 mg) was added slowly into the solution, and the mixture was kept at reflux with continuous stirring for 1 h. After the solution had cooled to room temperature colourless crystals appeared. The crystals were filtered and washed with methanol for three times. Recrystallization from an absolute methanol yielded block-shaped single crystals of the title compound.

### Refinement

The amide H atom was located in a difference map and its positional parameters were refined. All other H atoms were placed in calculated positions (C-H = 0.93 Å) and refined using a riding model. The *U*_iso_(H) values were set at 1.2*U*_eq_(C,N).

### Crystal data

**Table:**

C_14_H_10_BrN_3_O_3_	*Z* = 1
*M**_r_* = 348.16	*F*(000) = 174
Triclinic, *P*1	*D*_x_ = 1.654 Mg m^−^^3^
Hall symbol: P 1	Mo *K*α radiation, λ = 0.71073 Å
*a* = 4.8718 (17) Å	Cell parameters from 1163 reflections
*b* = 6.842 (2) Å	θ = 2.8–26.3°
*c* = 10.709 (4) Å	µ = 2.95 mm^−^^1^
α = 98.014 (5)°	*T* = 298 K
β = 93.258 (6)°	Block, colourless
γ = 97.413 (5)°	0.20 × 0.18 × 0.17 mm
*V* = 349.5 (2) Å^3^	

### Data collection

**Table:**

Bruker SMART CCD area-detector diffractometer	1998 independent reflections
Radiation source: fine-focus sealed tube	1584 reflections with *I* > 2σ(*I*)
graphite	*R*_int_ = 0.013
ω scans	θ_max_ = 30.7°, θ_min_ = 1.9°
Absorption correction: multi-scan (*SADABS*; Bruker, 2001)	*h* = −6→6
*T*_min_ = 0.590, *T*_max_ = 0.634	*k* = −8→8
2347 measured reflections	*l* = −11→15

### Refinement

**Table:**

Refinement on *F*^2^	Secondary atom site location: difference Fourier map
Least-squares matrix: full	Hydrogen site location: inferred from neighbouring sites
*R*[*F*^2^ > 2σ(*F*^2^)] = 0.027	H atoms treated by a mixture of independent and constrained refinement
*wR*(*F*^2^) = 0.064	*w* = 1/[σ^2^(*F*_o_^2^) + (0.0058*P*)^2^] where *P* = (*F*_o_^2^ + 2*F*_c_^2^)/3
*S* = 0.95	(Δ/σ)_max_ = 0.001
1998 reflections	Δρ_max_ = 0.22 e Å^−^^3^
193 parameters	Δρ_min_ = −0.23 e Å^−^^3^
3 restraints	Absolute structure: Flack (1983), 235 Friedel pairs
Primary atom site location: structure-invariant direct methods	Flack parameter: 0.021 (8)

### Special details

**Table:**

Geometry. All e.s.d.'s (except the e.s.d. in the dihedral angle between two l.s. planes) are estimated using the full covariance matrix. The cell e.s.d.'s are taken into account individually in the estimation of e.s.d.'s in distances, angles and torsion angles; correlations between e.s.d.'s in cell parameters are only used when they are defined by crystal symmetry. An approximate (isotropic) treatment of cell e.s.d.'s is used for estimating e.s.d.'s involving l.s. planes.
Refinement. Refinement of *F*^2^ against ALL reflections. The weighted *R*-factor *wR* and goodness of fit *S* are based on *F*^2^, conventional *R*-factors *R* are based on *F*, with *F* set to zero for negative *F*^2^. The threshold expression of *F*^2^ > σ(*F*^2^) is used only for calculating *R*-factors(gt) *etc*. and is not relevant to the choice of reflections for refinement. *R*-factors based on *F*^2^ are statistically about twice as large as those based on *F*, and *R*- factors based on ALL data will be even larger.

### Fractional atomic coordinates and isotropic or equivalent isotropic displacement parameters (Å^2^)

**Table:**

	*x*	*y*	*z*	*U*_iso_*/*U*_eq_	
Br1	0.28524 (6)	−0.18204 (5)	−0.03061 (4)	0.08281 (18)	
O1	0.9938 (4)	0.7003 (4)	0.2644 (2)	0.0599 (6)	
O2	0.1064 (8)	1.2188 (6)	0.7780 (3)	0.0860 (11)	
O3	0.2286 (10)	0.9674 (5)	0.6631 (3)	0.1064 (15)	
N1	0.5685 (5)	0.7559 (4)	0.3185 (2)	0.0399 (5)	
H1	0.394 (9)	0.725 (7)	0.293 (4)	0.048*	
N2	0.6709 (5)	0.9301 (4)	0.3980 (2)	0.0405 (5)	
N3	0.2485 (7)	1.1447 (5)	0.6997 (3)	0.0584 (8)	
C1	0.6174 (7)	0.4513 (5)	0.1848 (3)	0.0385 (7)	
C2	0.7401 (7)	0.3754 (5)	0.0775 (3)	0.0506 (8)	
H2	0.8910	0.4505	0.0495	0.061*	
C3	0.6386 (8)	0.1890 (6)	0.0126 (3)	0.0564 (9)	
H3	0.7180	0.1400	−0.0602	0.068*	
C4	0.4217 (8)	0.0767 (5)	0.0555 (3)	0.0504 (8)	
C5	0.2932 (7)	0.1486 (5)	0.1614 (3)	0.0447 (7)	
H5	0.1429	0.0722	0.1889	0.054*	
C6	0.3932 (6)	0.3357 (5)	0.2250 (3)	0.0420 (7)	
H6	0.3090	0.3855	0.2962	0.050*	
C7	0.7440 (6)	0.6473 (4)	0.2577 (3)	0.0404 (6)	
C8	0.4917 (6)	1.0171 (4)	0.4562 (3)	0.0384 (6)	
H8	0.3061	0.9609	0.4479	0.046*	
C9	0.5831 (6)	1.2100 (4)	0.5376 (3)	0.0393 (6)	
C10	0.4633 (7)	1.2766 (5)	0.6481 (3)	0.0433 (7)	
C11	0.5462 (8)	1.4635 (6)	0.7158 (3)	0.0607 (9)	
H11	0.4634	1.5029	0.7896	0.073*	
C12	0.7486 (9)	1.5906 (6)	0.6752 (4)	0.0660 (10)	
H12	0.8015	1.7178	0.7199	0.079*	
C13	0.8755 (8)	1.5290 (5)	0.5668 (3)	0.0550 (9)	
H13	1.0172	1.6139	0.5397	0.066*	
C14	0.7919 (8)	1.3419 (6)	0.4990 (3)	0.0489 (9)	
H14	0.8772	1.3031	0.4258	0.059*	

### Atomic displacement parameters (Å^2^)

**Table:**

	*U*^11^	*U*^22^	*U*^33^	*U*^12^	*U*^13^	*U*^23^
Br1	0.1255 (4)	0.04369 (19)	0.0679 (2)	−0.00267 (18)	0.00284 (18)	−0.01585 (13)
O1	0.0231 (11)	0.0608 (15)	0.0872 (15)	−0.0003 (10)	0.0026 (10)	−0.0128 (12)
O2	0.082 (2)	0.113 (3)	0.074 (2)	0.030 (2)	0.0420 (17)	0.0233 (19)
O3	0.149 (4)	0.068 (3)	0.087 (2)	−0.047 (2)	0.049 (2)	−0.0006 (17)
N1	0.0237 (12)	0.0358 (13)	0.0545 (13)	−0.0004 (10)	−0.0015 (10)	−0.0072 (11)
N2	0.0343 (13)	0.0331 (12)	0.0494 (12)	−0.0022 (10)	−0.0040 (10)	−0.0015 (10)
N3	0.057 (2)	0.068 (2)	0.0476 (14)	−0.0014 (16)	0.0036 (14)	0.0092 (14)
C1	0.0303 (16)	0.0361 (15)	0.0469 (15)	0.0039 (13)	−0.0061 (13)	0.0027 (12)
C2	0.0450 (19)	0.050 (2)	0.0551 (17)	0.0058 (15)	0.0104 (14)	0.0015 (15)
C3	0.067 (2)	0.057 (2)	0.0417 (15)	0.0094 (18)	0.0084 (15)	−0.0075 (14)
C4	0.062 (2)	0.0360 (16)	0.0488 (16)	0.0072 (15)	−0.0087 (15)	−0.0032 (13)
C5	0.0430 (18)	0.0365 (16)	0.0515 (16)	−0.0015 (13)	0.0030 (14)	0.0024 (13)
C6	0.0379 (17)	0.0418 (17)	0.0438 (15)	0.0048 (13)	0.0031 (13)	−0.0008 (13)
C7	0.0318 (16)	0.0389 (16)	0.0484 (14)	0.0018 (12)	−0.0009 (12)	0.0038 (12)
C8	0.0322 (15)	0.0356 (15)	0.0435 (14)	−0.0016 (13)	0.0004 (12)	−0.0006 (12)
C9	0.0385 (16)	0.0325 (15)	0.0444 (14)	−0.0001 (12)	−0.0029 (12)	0.0039 (12)
C10	0.0401 (17)	0.0418 (16)	0.0464 (14)	0.0022 (13)	0.0033 (13)	0.0042 (12)
C11	0.070 (2)	0.053 (2)	0.0542 (17)	0.0114 (19)	0.0013 (17)	−0.0095 (15)
C12	0.079 (3)	0.0351 (17)	0.075 (2)	−0.0046 (18)	−0.004 (2)	−0.0064 (16)
C13	0.058 (2)	0.0404 (19)	0.0614 (19)	−0.0124 (15)	0.0002 (17)	0.0091 (15)
C14	0.0495 (18)	0.041 (2)	0.051 (2)	−0.0091 (15)	0.0001 (17)	0.0075 (18)

### Geometric parameters (Å, °)

**Table:**

Br1—C4	1.897 (3)	C4—C5	1.387 (5)
O1—C7	1.219 (4)	C5—C6	1.377 (4)
O2—N3	1.216 (5)	C5—H5	0.93
O3—N3	1.211 (5)	C6—H6	0.93
N1—C7	1.341 (4)	C8—C9	1.478 (4)
N1—N2	1.382 (3)	C8—H8	0.93
N1—H1	0.87 (4)	C9—C14	1.391 (5)
N2—C8	1.264 (4)	C9—C10	1.395 (4)
N3—C10	1.474 (5)	C10—C11	1.378 (4)
C1—C6	1.389 (5)	C11—C12	1.360 (6)
C1—C2	1.393 (5)	C11—H11	0.93
C1—C7	1.493 (4)	C12—C13	1.386 (6)
C2—C3	1.380 (5)	C12—H12	0.93
C2—H2	0.93	C13—C14	1.380 (5)
C3—C4	1.364 (5)	C13—H13	0.93
C3—H3	0.93	C14—H14	0.93
			
C7—N1—N2	119.9 (2)	O1—C7—N1	122.7 (3)
C7—N1—H1	116 (3)	O1—C7—C1	121.2 (3)
N2—N1—H1	123 (3)	N1—C7—C1	116.0 (2)
C8—N2—N1	115.5 (2)	N2—C8—C9	118.6 (3)
O3—N3—O2	123.8 (4)	N2—C8—H8	120.7
O3—N3—C10	117.7 (3)	C9—C8—H8	120.7
O2—N3—C10	118.5 (4)	C14—C9—C10	116.6 (3)
C6—C1—C2	118.7 (3)	C14—C9—C8	118.6 (3)
C6—C1—C7	122.3 (3)	C10—C9—C8	124.7 (3)
C2—C1—C7	118.8 (3)	C11—C10—C9	121.9 (3)
C3—C2—C1	120.2 (3)	C11—C10—N3	117.4 (3)
C3—C2—H2	119.9	C9—C10—N3	120.7 (3)
C1—C2—H2	119.9	C12—C11—C10	120.3 (3)
C4—C3—C2	119.9 (3)	C12—C11—H11	119.8
C4—C3—H3	120.1	C10—C11—H11	119.8
C2—C3—H3	120.1	C11—C12—C13	119.5 (3)
C3—C4—C5	121.3 (3)	C11—C12—H12	120.3
C3—C4—Br1	120.4 (3)	C13—C12—H12	120.3
C5—C4—Br1	118.3 (3)	C14—C13—C12	120.1 (3)
C6—C5—C4	118.6 (3)	C14—C13—H13	119.9
C6—C5—H5	120.7	C12—C13—H13	119.9
C4—C5—H5	120.7	C13—C14—C9	121.5 (3)
C5—C6—C1	121.3 (3)	C13—C14—H14	119.2
C5—C6—H6	119.4	C9—C14—H14	119.2
C1—C6—H6	119.4		
			
C7—N1—N2—C8	−176.5 (3)	N2—C8—C9—C14	36.5 (4)
C6—C1—C2—C3	0.3 (5)	N2—C8—C9—C10	−147.7 (3)
C7—C1—C2—C3	175.9 (3)	C14—C9—C10—C11	0.1 (4)
C1—C2—C3—C4	−1.6 (5)	C8—C9—C10—C11	−175.8 (3)
C2—C3—C4—C5	2.2 (5)	C14—C9—C10—N3	−177.4 (3)
C2—C3—C4—Br1	−178.6 (3)	C8—C9—C10—N3	6.6 (4)
C3—C4—C5—C6	−1.4 (5)	O3—N3—C10—C11	−160.0 (4)
Br1—C4—C5—C6	179.4 (2)	O2—N3—C10—C11	18.4 (5)
C4—C5—C6—C1	0.1 (4)	O3—N3—C10—C9	17.7 (5)
C2—C1—C6—C5	0.4 (4)	O2—N3—C10—C9	−163.9 (3)
C7—C1—C6—C5	−175.0 (3)	C9—C10—C11—C12	0.6 (5)
N2—N1—C7—O1	−3.6 (4)	N3—C10—C11—C12	178.3 (4)
N2—N1—C7—C1	173.8 (2)	C10—C11—C12—C13	−1.5 (6)
C6—C1—C7—O1	143.2 (3)	C11—C12—C13—C14	1.6 (6)
C2—C1—C7—O1	−32.3 (4)	C12—C13—C14—C9	−0.8 (6)
C6—C1—C7—N1	−34.3 (4)	C10—C9—C14—C13	0.0 (5)
C2—C1—C7—N1	150.3 (3)	C8—C9—C14—C13	176.1 (3)
N1—N2—C8—C9	−176.7 (2)		

### Hydrogen-bond geometry (Å, °)

**Table:**

*D*—H···*A*	*D*—H	H···*A*	*D*···*A*	*D*—H···*A*
N1—H1···O1^i^	0.900 (11)	1.909 (19)	2.791 (3)	166 (6)

Symmetry codes: (i) *x*−1, *y*, *z*.
